# Efficacy and side effects of bio-fabricated sardine fish scale silver nanoparticles against malarial vector *Anopheles stephensi*

**DOI:** 10.1038/s41598-021-98899-5

**Published:** 2021-10-01

**Authors:** Kadarkarai Murugan, Jayapal Subramaniam, Rajapandian Rajaganesh, Chellasamy Panneerselvam, Pandiyan Amuthavalli, Murugan Vasanthakumaran, Sudalaimani Jayashanthini, Devakumar Dinesh, Jaganathan Anitha, Lan Wang, Jiang-Shiou Hwang, Hans-Uwe Dahms, Sunaina Mudigonda, Al Thabiani Aziz

**Affiliations:** 1 Division of Entomology, Department of Zoology, School of Life Sciences, Bharathiar University, Coimbatore, Tamil Nadu 641046 India; 2 Department of Biology, Faculty of Science, University of Tabuk, Tabuk, 71491 Saudi Arabia; 3 Department of Zoology, Kongunadu Arts and Science College, Coimbatore, 641029 India; 4 School of Life Science, Shanxi University, Taiyuan, 030006 Shanxi China; 5 Institute of Marine Biology, National Taiwan Ocean University, Keelung, 20224 Taiwan; 6 Center of Excellence for Ocean Engineering, National Taiwan Ocean University, Keelung, 20224 Taiwan; 7 Center of Excellence for the Oceans, National Taiwan Ocean University, Keelung, 20224 Taiwan; 8 Department of Biomedical Science and Environmental Biology, Kaohsiung Medical University, Kaohsiung, 80424 Taiwan; 9 Research Center for Environmental Medicine, Kaohsiung Medical University, Kaohsiung City, 807 Taiwan; 10 Department of Marine Biotechnology and Resources, National Sun Yat-Sen University, Kaohsiung City, 804 Taiwan; 11 Department of Medicinal and Applied Chemistry, Kaohsiung Medical University, Kaohsiung City, 807 Taiwan

**Keywords:** Biological techniques, Biotechnology, Environmental sciences, Medical research

## Abstract

Mosquitoes are a great menace for humankind since they transmit pathogenic organisms causing Malaria, Dengue, Chikungunya, Elephantiasis and Japanese encephalitis. There is an urgent need to discover new and novel biological tools to mitigate mosquito-borne diseases. To develop bioinsecticides through newly developed nanotechnology is another option in the present research scenario. In this study we synthesize and characterize sardine fish scales with silver nitrate by adopting various instrumental techniques such as UV- and FTIR-spectroscopy, energy-dispersive X-ray (EDAX), X-ray diffraction analyses (XRD) and scanning electron microscopy (SEM). Toxicity bioassays were conducted with young developmental stages of mosquito vectors. Significant mortality appeared after different life stages of mosquito vectors (young larval and pupal instars were exposed to the nanomaterials). LC_50_ values were 13.261 ppm for young first instar larvae and 32.182 ppm for pupae. Feeding and predatory potential of *G. affinis*, before and after exposure to nanoparticles against mosquito larval (I & II) instars of the mosquitoes showed promising results in laboratory experiments. Feeding potential of mosquito fish without nanoparticle treatment was 79.7% and 70.55% for the first and second instar larval populations respectively. At the nanoparticle-exposed situation the predatory efficiency of mosquitofish was 94.15% and 84.3%, respectively. Antioxidant enzymes like (SOD), (CAT), and (LPO) were estimated in the gill region of sardine fish in control and experimental waters. A significant reduction of egg hatchability was evident after nanoparticle application. It became evident from this study that the nano-fabricated materials provide suitable tools to control the malaria vector *Anopheles stephensi* in the aquatic phase of its life cycle. This finding suggests an effective novel approach to mosquito control.

## Introduction

Malarial fever is the most important infectious disease around the tropical belt worldwide. Several biotic and abiotic factors favour the spread of this disease^1^. The mosquito species *Anopheles stephensi* is transmits the protozoan parasite *Plasmodium falciparum* that causes malaria and this provides severe problems in Asian countries^2,3^ as well as elsewhere in the tropics. Allelochemicals (plant secondary metabolites) are a rich source of biologically active molecules’ which are important for Ayurveda, Unani, Siddha medicines and other therapeutic systems^4,5^. In this study, we prepared an eco-friendly mode and silver nanoparticles from fish scale extract acting as a reducing/stabilizing agent. Other abiotic parameters like temperature, pH on the quantification of the materials on the biosynthesis of silver nanoparticles were investigated^6^. Nowadays, silver nanomaterials show a variety of biological activities against microbial technology and nanomedical applications^7,8^. So, AgNP has several different medical uses and it has several potential uses in the field of cosmetics^9^ which includes mosquito vector control^10–12^. Generally, silver-nanoparticles are widely used for microbe suppression programs, hence, it can be employed for the control of medical problems^13,14^. For integrated vector control programs in aquatic systems, biocontrol through predation provides an important regulation tool within ecosystems^15,16^. Predatory fishes are commonly employed for mosquito larval control in cultivated paddy fields, wetlands, as well as aquaculture ponds^17,18^.

A detailed investigation was made by Murugan et al.^19^ on the predatory behavior of gold NPs and guppies against young larval instars of mosquitoes. Due to the negative impacts of insecticidal synthetic chemicals on the environment, we have investigated the toxicological, ecological and physiological status of animals and their mode of action being of paramount importance^20,21^. Enzymes are involved in regulating the biological functions of living organisms^22,23^. Environmental contaminants might affect metabolic and physiological processes at the same time they form residues which are absorbed in different tissues of water-dwelling organisms, including fishes^24^. Pollutants commonly accumulate in the tissues and organs such as in the liver, gill and blood tissues. Pollutants are the main causes of stress to particular organisms and fish have the ability to regulate metals to cope with temperature tolerance and to prevent toxic effects on the organisms. Furthermore, fish have innate mechanisms to detoxify toxic chemicals. In fish the gills are pivotal organs and perform various physiological activities such as respiration, acid–base regulation and nitrogenous waste elimination. To prevent ROS-mediated cellular damage, organisms generally activate antioxidant defense systems^25,26^. These include the two major enzymatic profile groups superoxide dismutase (SOD) and catalase (CAT) which are the most sensitive enzyme groups for free radical scavenging processes^3,27^. Damages caused by ROS are provided by lipid membrane oxidation and lipid peroxidation (LPO)^23,28^. Livingstone et al.^29^ studied various contaminants exposed to the production and oxidative damage in aquatic organisms. The toxicity, behavior and fate of nano-pesticides were less investigated^30,31^.

**Figure 1:**
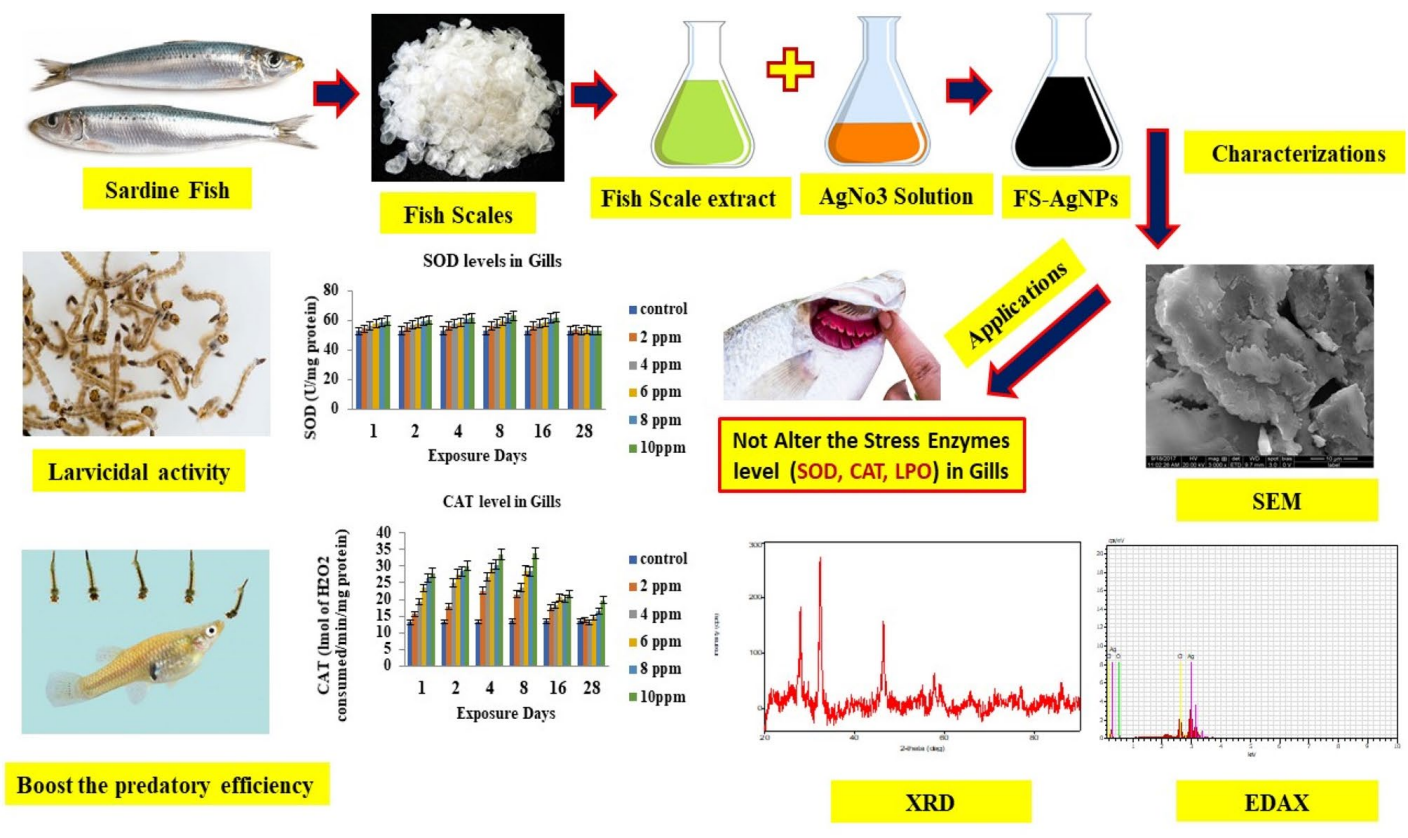
Pictorial representations of the salient steps of this study.

In this paper, we used bio-fabricated silver nanomaterials from fish scales (Fig. 1). These materials were nano-characterized by adopting various instrumentation techniques for the following issues: (i) Toxicity effect of biosynthesized fish scale-nanomaterials on the larvae and pupae of *An. stephensi* and also studied the life history performance and ovicidal effect; (ii) predatory potential of fish on mosquito larvae in contaminated environments; (iii) impact of bio-fabricated fish nanomaterials on the concentrations of superoxide dismutase, catalase and lipid peroxidase enzymes in the gill region of the teleost fish *G. affinis.*

## Materials and methods

We confirm that all experimental protocols were approved by Institutional Animal Ethical Committee, Bharathiar University, Coimbatore, India.

### Fish scale extraction procedure

Fish scale materials (*Sardinella longiceps*) were procured from the fish market, Ukkadam, Coimbatore, India. Fish scales were transferred to the Department of Zoology, Bharathiar University, were washed thoroughly for discarded abdicable wreckages, air-dried, and stored at − 60 ℃ while waiting for the next process for no more than 30 days; AgNO_3_ (silver nitrate ≥ 99.0% pure- ≤ 0.01% not HCl), SOD, CAT, and LPO enzyme analytical kits and other chemicals were purchased from Sigma Chemicals in the USA.

### Bio-synthesis and characterization of fish scale bio-fabricated silver nanoparticles (SFS-AgNPs)

For the synthesis of bio-fabricated fish scale silver nanoparticles, we adopted the procedure of Murugan et al.^19^ which was previously described and slightly modified by Muthumari et al.^32^ and Dinesh et al.^33^. Initially, the Sardine Fish Scale extract was made by adding 10 g of SFS-grains to 100 mL double distilled water which was then stewed at 60 ℃ for 20 min in the filtered extract used to prepare the SFS-AgNPs. The filtered SFS aqueous extract of 10 mL with different concentrations (10%, 20%, 30% and 40%) was added separately to 10 mL of 1 mM AgNO_3_ aqueous solution and boiled at 70 ℃ for 10 min followed by incubation at room temperature for cooling the solution^19^. After 24 h the solution of dark brown sediments indicated the formation and presence of the AgNPs. Then physiochemical characterizations were done viz., UV–vis, SEM, EDX, FTIR and XRD analyses^34^. SFS-AgNPs were authenticated by UV–vis, followed by SEM, EDX, FT-IR and XRD. UV–vis spectrophotometry enables the user to illustrate the shape and size of nanofabricated materials in water solution^35^.

### Mosquitocidal properties of SFS-AgNPs on the malarial vector *A. stephensi *L.

The eggs and young larval and pupal mosquitoes were maintained and cultured and pathogen-free colonies were maintained at laboratory conditions (27 ± 2 °C, 75–85% R.H. and 14 h:10 h (L:D) photoperiod) following Subramaniam and Murugan^36^ and Subramaniam et al.^37,38^. We have followed the procedure by Murugan et al.^10,39,40^ where five replications were made. In each replicate, there were twenty-five larvae/pupae in the two hundred fifty millilitre of double distilled water including the required concentration of sardine fish scale extract (ppm) and SFS-AgNPs (ppm), respectively. Ovicidal efficiency was elaborated using methods by Mullai and Jebanesan^41^ and Panneerselvam and Murugan^42^. Hundred eggs were used for each replicate and five replications were made at different concentrations of SFS extract (50, 75, 100, 125 and 150 ppm) and SFS-AgNPs (10, 20, 30, 40 and 50 ppm). After 98 h post-treatment, egg mortality (no hatching and closed opercula) was calculated following Kovendan et al.^43^.

### Predatory potential of *G. affinis*

*G. affinis* was maintained in the laboratory in a definite size of the fish tank and cultured fishes were used to test the predatory potential of mosquitofish. Different larval stages (I-IV) of *A. stephensi* were used for a predatory bioassay by Murugan et al.^11,44^ and Subramaniam et al.^45^. The feeding potential of the fishes was recorded with the decrease in doses for I-IV larvae of *A. stephensi* i.e. 1/3 of the LC_50_ values of SFS extract (Table 1) and SFS-AgNPs (Table 2). Experiments were replicated five times and larvae were replaced daily and the experiments were monitored periodically (predatory/prey potential) the predatory efficiency was calculated by using formula originally defined by Murugan et al.^19,34^ and Subramaniam et al.^46,47^.

**Table 1:** Larval and pupal toxicity of sardine fish scales extract against *Anopheles stephensi.*

Larval instars	LC_50_ (LC_90_)	95% confidence limit LC_50_ (LC_90_)		Regression equation	*χ*^2^ (*d.f.* = 4)
		Lower	Upper		
1st instar	80.923 (173.939)	71.804 (157.103)	89.418 (198.695)	*y* = 1.115 + 0.014*x*	0.819 n.s.
2nd instar	102.623 (224.472)	91.768 (195.184)	115.116 (273.433)	*y* = 1.079 + 0.011*x*	0.818 n.s.
3rd instar	151.609 (344.288)	129.004 (269.691)	197.958 (521.433)	*y* = 1.008 + 0.007*x*	0.016 n.s.
4th instar	170.382 (336.187	146.134 (269.395)	218.930 (481.779)	*y* = 1.317 + 0.008*x*	2.902 n.s.
Pupa	215.561 (411.677)	174.790 (310.807)	321.136 (684.715)	*y* = 1.409 + 0.007*x*	0.741 n.s.

No mortality was observed in the control.

LC_50_: lethal concentration was killing half of the treated organisms, LC_90_: lethal concentration was killing  90% of the treated organisms, χ^2^: Value of the chi-square, *d.f.*: degree of freedom, n.s.: non-significant (α = 0.05).

**Table 2:** Larval and pupal toxicity of AgNP synthesized from sardine fish scales against *Anopheles stephensi.*

Larval instars	LC_50_ (LC_90_)	95% confidence limit LC_50_(LC_90_)		Regression equation	*χ*^*2*^ (*d.f.* = 4)
		Lower	Upper		
Larva I	13.261 (25.149)	11.607 (23.483)	14.587 (27.406)	*y* = 1.430 + 0.108*x*	0.247n.s.
Larva II	16.227 (28.533)	14.857 (26.660)	17.429 (31.080)	*y* = 1.690 + 0.104*x*	1.821n.s.
Larva III	19.300 (34.083)	17.889 (31.399)	20.664 (37.964)	*y* = 1.673 + 0.087*x*	1.954n.s.
Larva IV	22.855 (38.269)	21.430 (34.960)	24.478 (43.195)	*y* = 1.900 + 0.083*x*	2.437n.s.
Pupa	32.182 (56.822)	28.742 (47.631)	38.427 (75.093)	*y* = 1.674 + 0.052*x*	0.243n.s.

No mortality was observed in the control.

LC_50_: lethal concentration was killing half of the treated organisms, LC_90_: lethal concentration was killing 90% of the treated organisms, χ^2^: Value of the chi-square, *d.f.*: degree of freedom, n.s.: non-significant (α = 0.05).

### Collection and preparation of samples for enzyme assays

At the completion of each experimental period (1,2,4,8,16 and 28 days) fishes from control, Sardine fish scale extract and sardine fish scale silver nanoparticles were used for treatments (treatment I, II and III) at desired concentrations (2,4,6,8 and 10 ppm) respectively. Blood samples of organs (gills) were collected and stored at cold conditions. The tissues were used to evaluate enzymological parameters (LPO, SOD and CAT). Lipid peroxidation (LPO) was investigated following the methods described and modified by Gupta and Verma^48^. Chemical concentrations were estimated by the procedure of Chen et al.^49^. CAT activities were studied after Bao et al.^50^. Enzyme activation was estimated from the gill region of *G. affinis* after the treatment of fish scale biofabricated silver nanoparticles at different concentrations viz. 2, 4, 6, 8, 10 ppm concentration after different periods (i.e. 1, 2, 4, 8, 16 days respectively). The following enzymes, SOD, CAT and LPO were estimated in the gill region after the exposure of nanoparticles at different dose levels.

### Data analysis

Lethal concentrations (LC_50_ and LC_90_) were determined by probit analysis using mortality data^51^. The SPSS statistical package 16.0 version was used for all sample analysis calculations. All data were subjected to Analysis of variance. The means were separated using Duncan’s multiple range test modified by Alder and Rossler^52^. Antioxidant enzyme studies SOD and calculated data were shown as means ± SE, percentage changes were compared by the means of treated data against their controls. Data were considered significant at (P < 0.01) and (P < 0.05) levels.

We wish to confirm that all methods were carried out in accordance with relevant guidelines and regulations of Bharathiar University, India and for the manuscript entitled “Efficacy of bio-fabricated Sardine fish scale silver nanoparticles against malarial vector and their effect on antioxidant enzymes of mosquito fish, *Gambusia affinis*” which was submitted to Scientific Reports.

We are confirming that the study was carried out in compliance with the ARRIVE guidelines for the experimentations and further experimental protocol framed by the of Bharathiar University, India and for the manuscript entitled “Efficacy of bio-fabricated Sardine fish scales silver nanoparticles against malarial vector and their effect on antioxidant enzymes of mosquito fish, *Gambusia affinis*”, which was submitted to Scientific Reports.

## Results and discussion

### Physicochemical characterization of nanoparticles by instrumentation techniques

Nanoparticles mediated with the aid of nanobiotechnology have provided a series of purposes viz., electronics, physics, drug delivery, stem cell therapy^16^. Nanostructured materials and its possessions must be examined in advanced practice. Regularly to designate the physicochemical representation of the unknown NPs, several corresponding techniques should promptly be employed (i.e. SEM, EDAX, FT-IR spectroscopy and X-ray diffraction studies)^6,53^ as we did in the present study.

**Figure 2:**
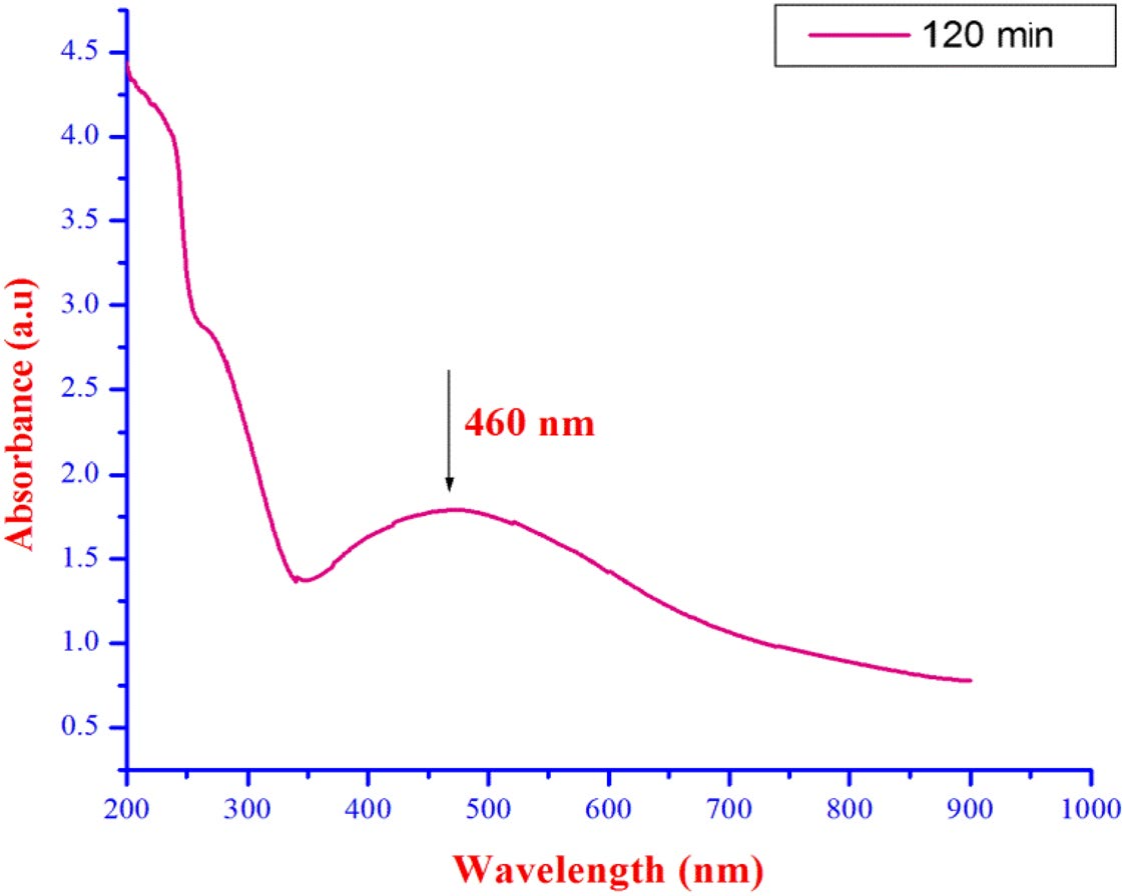
UV-vis spectra of the sardine fish scales fabricated AgNPs at 120 mins.

The reaction time of fish scale extract was visualized in the UV-visualization spectrum and provided in Fig. 2. The color intensity of the SFS extract incubated with AgNO_3_ solution at the start of the treatment and after 120 min of reaction is providing a peak at 460 nm (Fig. 2). Similarly, biofabrication of AgNPs was determined via a chromatic indicator represented by color change of the reaction substrate, from light green to brown which showed the biotransformation of Ag^+^ ion to Ag^0^^54,55^. The UV–vis spectra of fish scale extracts as a function of reaction time provided a strong resonance at about 410 nm which increased in intensity with time. The physically powerful Plasmon resonance centering at 400 nm increased in strength with time. This is explained with the excitation of longitudinal Plasmon vibrations of AgNPs in solution^56^.

**Figure 3:**
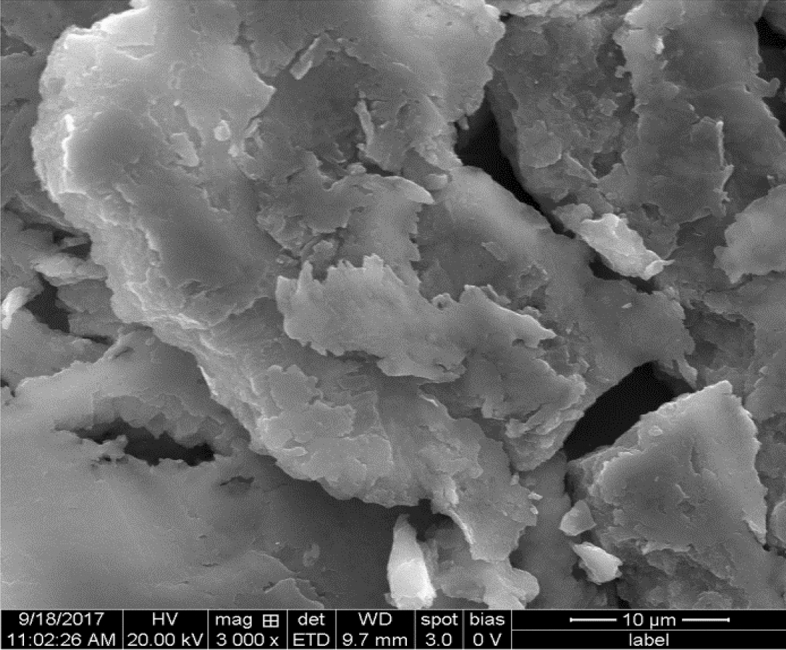
Scanning electron micrograph (SEM) of the sardine fish scales fabricated silver nanoparticles.

Scanning electron microscope (SEM) was used to characterize the morphology of the AgNPs and to observe the dissimilarities in size, capability for accumulation, and stability of experimental conditions^57^. The physical structure of AgNps was visualized by adopting the SEM analysis which procured hexagonal nanostructures as presented in Fig. 3. Santhosh Kumar et al.^58^ demonstrated by SEM that the morphologies of Ag-Nps were unique and uniform in size from 25 to 80 nm. Our results agreed with Murugan et al.^14^ that the visual part of morphology of chitosan-fabricated AgNP was studied by FE-SEM and showed spherically-shaped particles ranging from 30 to 50 nm.

**Figure 4:**
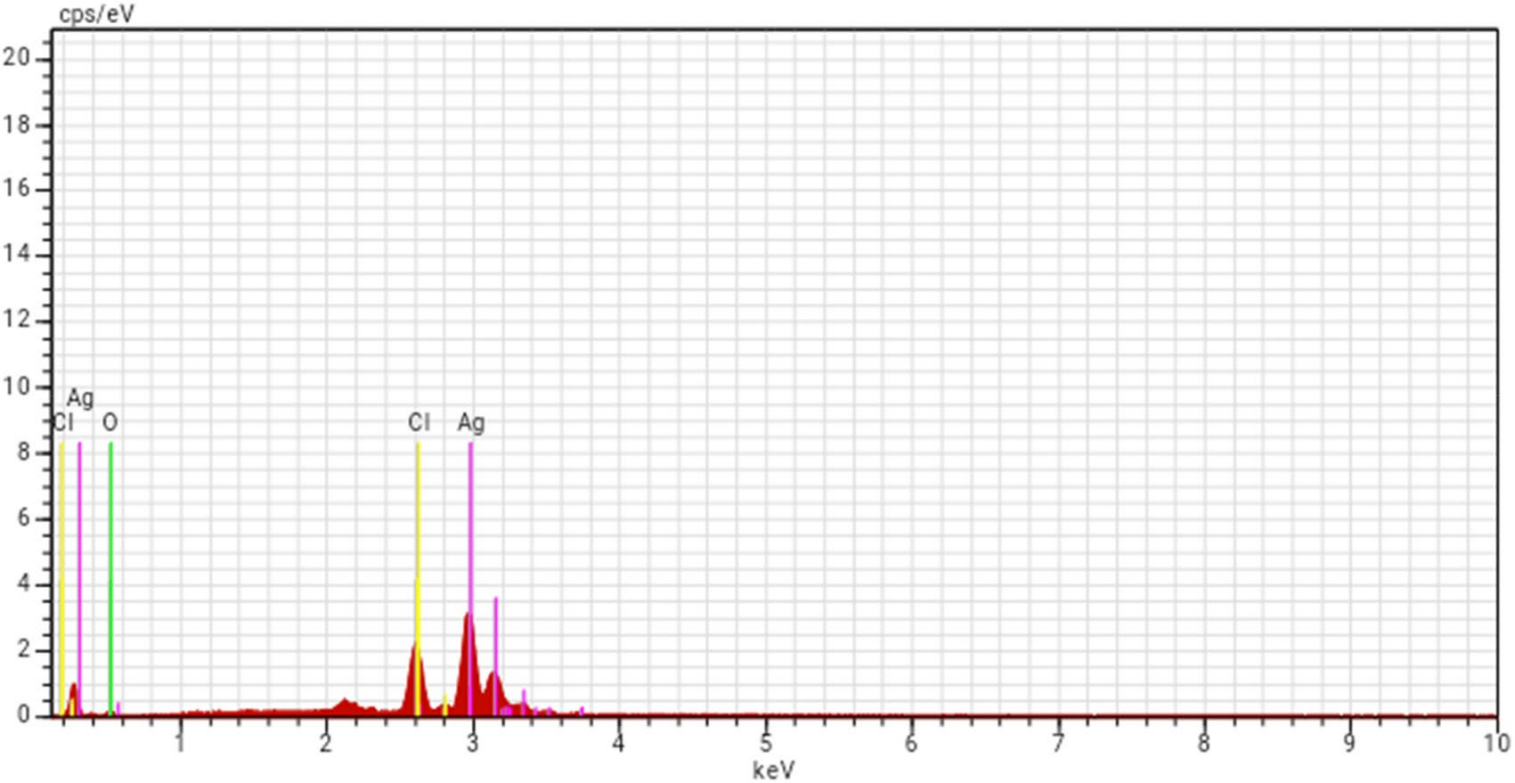
Energy-dispersive X-ray spectrum (EDX) of AgNPs fabricated sardine fish scale extract.

EDX spectra provided quantitative evidence and the occurrence of elements involved in the materialization of SFS-AgNPs synthesized at 25 °C and 80 °C **(**Fig. 4**)**. The silver atoms in the SFS-AgNPs and those from oxygen, silver and chloride provided strong signals. Our results agree with Murugan et al.^19^ who showed that metallic silver could be confirmed by strong silver signals. The results of Energy-dispersive X-ray (EDX) spectra provided evidence from *Centroceras calvulatum* extract and *Sonneratia alba* synthesized AgNPs, respectively^10,21^.

**Figure 5:**
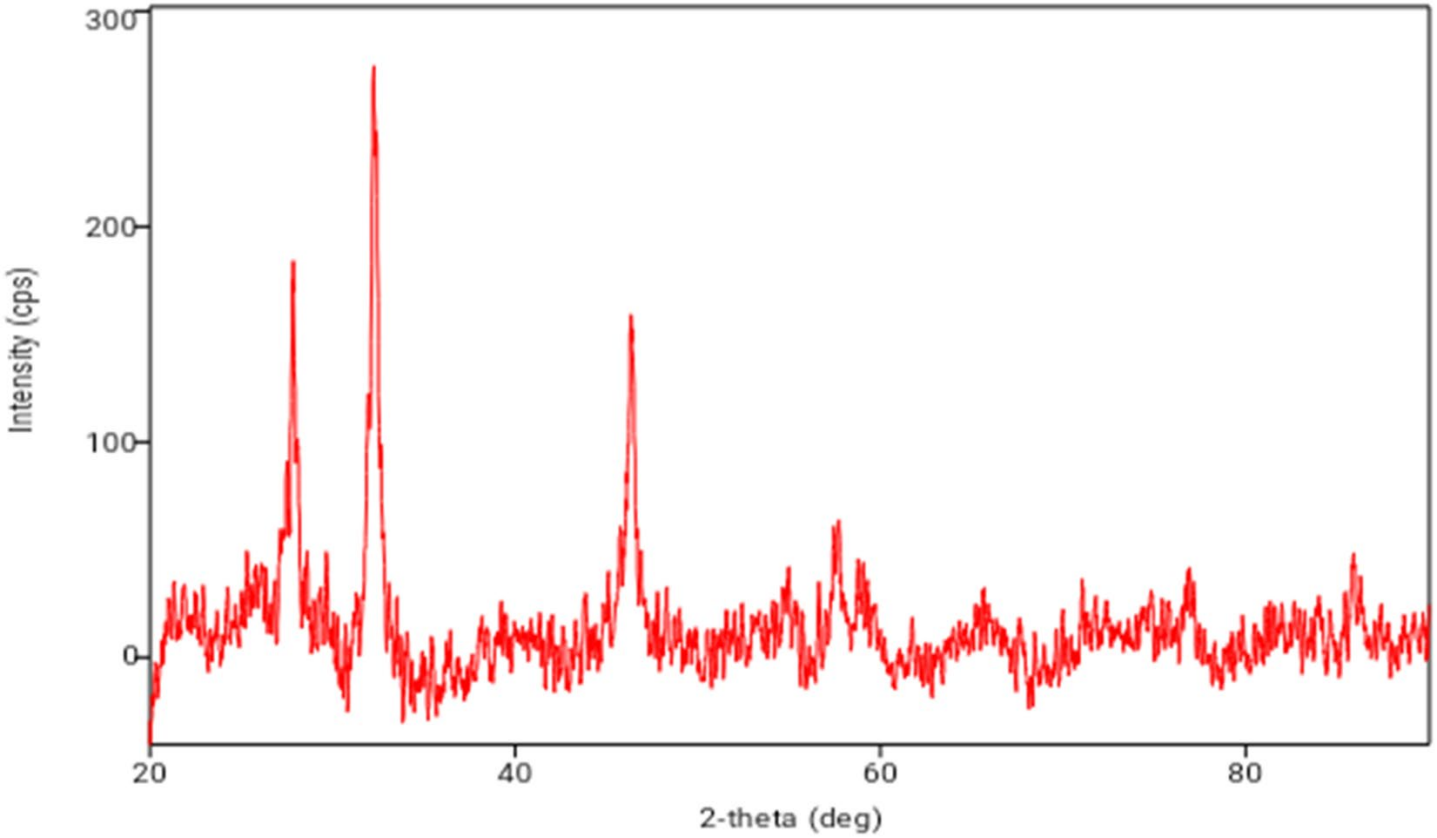
XRD pattern of the sardine fish scales fabricated silver nanoparticles.

The structure and crystal lattice of the SFS-AgNPs (Sardine fish scale synthesized silver nanoparticles—see Fig. 5) are categorized and authenticated by employing X-ray powder diffraction (XRD) pattern of SFS-AgNPsps. The Braggs reflections (111, 200, 220 and 311) were noticed in the XRD pattern at 2 theta 28.20°, 32.45°, 46.50° and 58.60° results, corresponding to the face centered cubic structure, advocating that crystalline in nature and bioorganic junctures occur on the surface of the SFS-AgNPs. These data are in concurrence with earlier investigations. Anal and Kamal^59^ reported about the biosynthesis of silver nanomaterials by employing fish processing waste. A XRD study indicated the occurrence of broad peaks of nanoparticles that were of very small size and confirmed their semi-crystalline nature. Parthiban et al.^60^, confirmed the crystalline nature and showed that the XRD pattern intensities of silver nanoparticles from *Annona reticulata* had three dispersion peaks with 2 theta values, at 37.56°, 43.25° and 64.10° (111, 200 and 220) respectively^40,61^.

**Figure 6:**
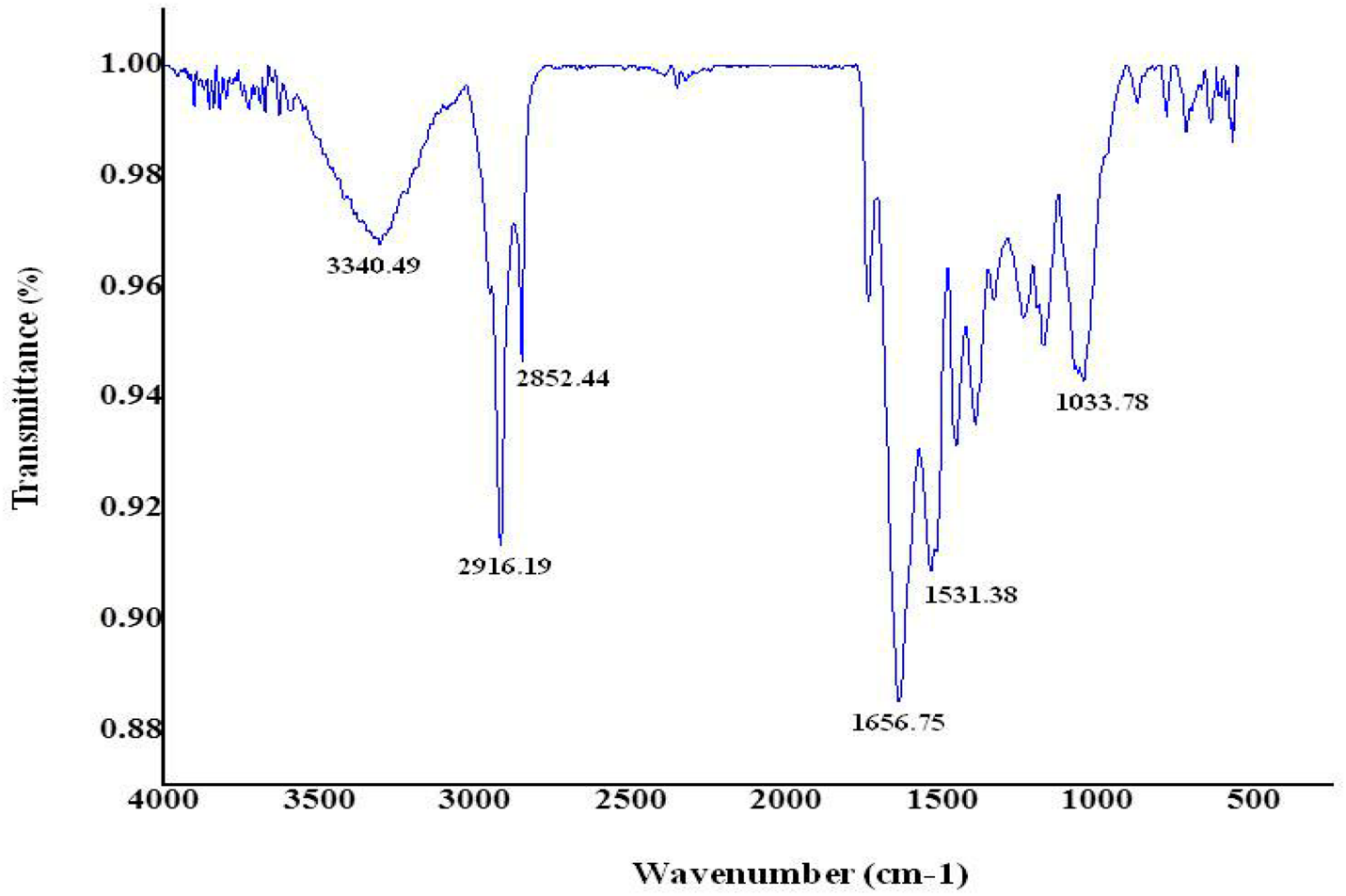
Fourier transform infrared (FTIR) spectrum of AgNPs fabricated sardine fish scale extract.

Fourier transformed infrared spectroscopy (FTIR) is another technique to determine physiochemical parameters and elucidate the structure of nanomaterials, based on the electromagnetic wavelength within the mid-infrared region (4000–400 cm^−1^)^62^. The FTIR analysis represents a non-invasive, value added, economical and simple method to classify the function of biological elements in the contraction of silver nitrate to silver^63^. FTIR spectrum analysis could be applied to confirm the interaction among the presence of amine groups in the combined nanostructures of sardine fish scale extract and silver nitrate. Figure 6 shows spectral maxima that reveal carbonyl groups such as from polyphenols such as N–O stretching (nitroxide compound), S=O stretching (sulfoxide), C=C stretching (alkene), C=O stretching (carboxylic), C–H stretching (alkene), N–H stretching (secondary amine), respectively. Altogether the data of the present study indicate that particles secured by Ag Nanomaterials had free and bound alkane and amine groups^64^.

Similarly, many researchers have studied that biofabricated nanoparticles are possessing different functional groups, including alkene and alkane^65,66^, methylene^67^, aromatic amine^68^, and carboxylic acid^33^, carboxyl groups or/in amide groups^67^ were revealed in earlier studies as reducing agents in the biofabrication of AgNPs.

### Nanoparticles as mosquito larvicides

Sardine fish scale extract and its biosynthesized silver nanoparticles with various concentrations expressed their toxicity against young instars and pupal populations of the malarial vector. However, the toxic effect was significantly higher in biosynthesized silver nanoparticles and these results are shown in Tables 1 and 2. For example, in the case of fish scale extract treatment of the fourth instar larvae, its LC_50_ was 170.38 ppm and for fish scale biosynthesized silver nanoparticles the LC_50_ was 22.85 ppm, respectively. Moreover, most of the biogenic nanostructures are spherical in shape^69^ with only some exceptionally being of oval shape^70^. Similar to earlier studies, the fish scale nanoparticles in the present study are also spherical and provided greater larval and pupal toxicity to mosquitoes^40^.

### Nanoparticles as mosquito ovicides

Sardine fish scale extract and sardine fish scale biosynthesized nanoparticles greatly affected the hatchability of eggs of mosquito vectors (Table 3). There was no hatching (NH) occurring at 150 ppm sardine fish scale extract whereas the sardine fish scale biosynthesized nanoparticles provided no hatchability (NH), even at the lowest dose group of 40 ppm. Madhiyazhagan et al.^71^ conducted experiments against malarial, filarial, and dengue vector and *Sargassum muticum*-fabricated silver nanostructures with a single dose (30 μg/mL) expressing significant ovicidal activities. Furthermore, Rajaganesh et al.^72^ found that 25 μg/mL administration led to 100% inhibition of hatchability by the exposure to fern (*Dicranopteris linearis*)-fabricated nanostructures.

**Table 3:** Ovicidal activity of sardine fish scales extract against the malarial vector *Anopheles stephensi*.

Treatment	Egg hatchability (%)							
	Concentration (ppm)							
	Control	50 ppm	75 ppm	100 ppm	125 ppm	150 ppm	Regression equation	*χ*^2^ (*d.f.* = 4)
Sardine fish scales extract	80.4 ± 1.34^a^	63.8 ± 1.48^b^	52.0 ± 1.87^c^	33.4 ± 1.14^d^	25.2 ± 0.83^e^	NH	*y* = 1.469 + 0.020*x*	14.87

	Control	10 ppm	20 ppm	30 ppm	40 ppm	50 ppm		
Sardine fish scale extract-AgNPs	92.4 ± 0.54^a^	53.2 ± 1.09^b^	41.8 ± 1.30^c^	23.4 ± 0.89^d^	NH	NH	y = 0.940 + 0.066x	15.50

NH-No hatchability (100% mortality).

Means followed by the same letter are not significantly different (P < 0.05).

### Predatory efficiency of *G. affinis* combined with SFS extract and SFS-AgNPs against *A. stephensi*

Predatory fish showed a high predation activity against malarial vectors and the first instar larvae were the most preferred food for the mosquito fish, *G. affinis.* In our predation experiments, predatory efficiency was 79.70% (first instar), 70.55% (second instar), 62.75% (third instar) and 46.30% (fourth instar), respectively (Table 4). Notably, predatory efficacy of *G. affinis* after application of silver nanoparticles at their elevated dose level reached 94.15%, 84.30%, 71.05% and 56.50%, from first to fourth instar larvae, respectively (Table 4).

**Table 4:** Predatory efficiency on mosquito *A. stephensi* by the teleost fish *Gambusia affinis*.

Treatment	Target	Day light time (n)	Night time (n)	Total predation nos	Percentage of predation
Standard conditions	I instar	174.0 ± 1.22^d^	144.8 ± 2.68^ cd^	*318.8*	79.7^d^
	II instar	158.8 ± 0.83^c^	123.4 ± 1.51^c^	282.2	70.55^c^
	III instar	138.6 ± 0.54^b^	112.4 ± 1.81^b^	251.0	62.75^b^
	IV instar	097.2 ± 2.04^a^	088.0 ± 1.22^4a^	185.2	46.3^a^
Post-treatment with AgNPs	I instar	193.6 ± 1.14^d^	183.0 ± 1.22	*376.6*	94.15^d^
	II instar	172.6 ± 0.89^bc^	164.6 ± 1.51^c^	337.2	84.3^c^
	III instar	155.8 ± 1.64^b^	128.4 ± 0.89^b^	284.2	71.05^b^
	IV instar	122.6 ± 1.14^a^	103.4 ± 1.51^a^	226.0	56.5^a^

Predation rates are represented by means ± SD of four replicates (1 fish vs 200 mosquitoes per replicate).

Control was clean water without predators within each column, means followed by the same letter are not.

Significantly different (P < 0.05).

### Effect of nanoparticles against non-target aquatic organisms

Silver nanoparticles were tested before for their acute toxicity^55^. Most of the biosynthesized silver nanoparticles did not show any toxicity against any aquatic organism^73^. For instance, plant synthesized silver nanoparticles did not show any toxic impact compared to fabricated nanomaterials to the predatory fish *Poecilia reticulata* after an exposure for 48 h. Earlier, Murugan et al.^21^ reported that a treatment with *S. alba* synthesized AgNP increased neither the consumption of predatory fish nor of guppy fish (*Poecilia reticulata*) for mosquito larvae.

### Antioxidant enzyme response to nanoparticle contaminated environments for non-target mosquito fish

**Figure 7:**
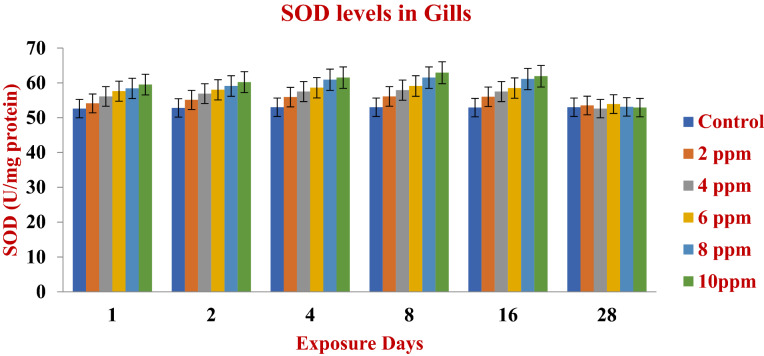
SOD enzymes in the gill region after the exposure (1–28 days) to AgNPs at different doses.

After exposure of fish to different doses of fish scale biofabricated silver nanoparticles there was a changing profile of enzyme activities in the gill region of the fish. Sodium dismutase (SOD) is a oxyradical scavenger and is mainly involved in the catalytic activity and liable for dismutation of exceedingly abundant superoxide radicals. A significant increase in the activity of SOD was noted (Fig. 7). This may be due to the synthesis of newer enzymes in the body system or enrichment of already existing enzymes loaded in the tissues at different concentrations. Interestingly, the level initially increased during 1, 2, 4 and 8 days and its level has been resumed very near to the uncontaminated biofabricated nanoparticles of fish scales. A similar study was conducted after the treatment of Cu nanoparticles at 25.6 μg L^−1^ after a 24 h treatment of gills of the mytilid clams *B. azoricus*^74^ and *M. galloprovincialis* after exposure to 5–25 μg L^−1^ Cu after 7 day treatment^75^.

**Figure 8:**
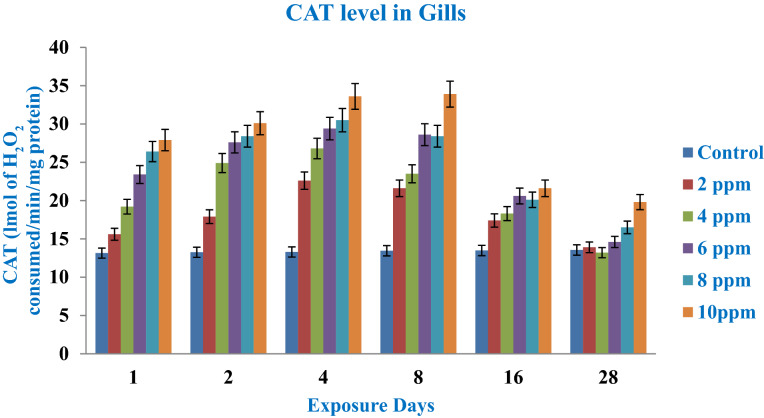
CAT enzymes in the gill region after the exposure (1–28 days) to AgNPs at different doses.

Catalase (CAT) activation provides another stress/antioxidant system of animals. This enzyme converts the free radicals H_2_O_2_ to H_2_O and O_2_ in the animal defense system. In our experiment after exposure to fish scale fabricated nanoparticles at 2, 4, 6, 8 and 10 ppm, the antioxidant enzyme (CAT) had slightly increased its activities up to day 1, 2 and 4 where it showed considerably elevated activities **(**Fig. 8). Yilmaz et al.^76^ reported peroxisome distributions in the gill region and its functional role on molecular H_2_O_2_ which is metabolized to molecular O_2_ and H_2_O.

**Figure 9:**
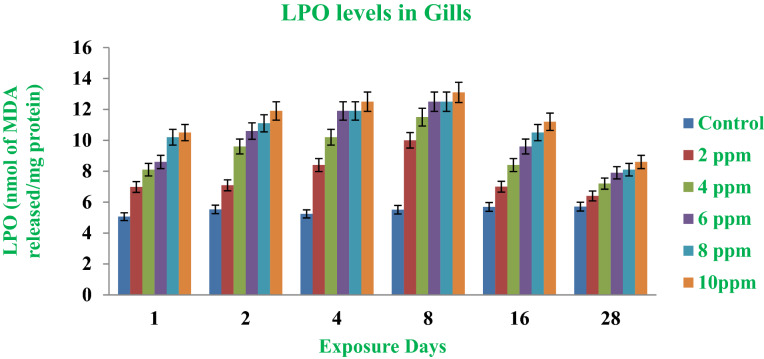
LPO enzymes in the gill region after the exposure (1–28 days) to AgNPs at different doses.

LPO is yet another very important indicator of oxidative enzymes in the cellular metabolism and it is the end product or outcome of oxidative deterioration of membrane lipids. LPO serves as a marker or oxidative enzyme and it has been extensively used as a biomarker for cellular activities^77^. Normally this enzyme (LPO) is evaluated/estimated by measuring the concentration of malondialdehyde (MDA) (Fig. 9). The higher activities and over-accumulation of the enzyme malondialdehyde not only damages cells but also triggers the process of apoptosis^78^. In the current study there was no change in the gills after nanoparticle exposure. However, a significant elevation of this enzyme was noted after 1, 2, 4 and 8 days.

## Conclusion

In conclusion, Sardine fish scale fabricated nanomaterials are hydrophilic, can uniformly disperse in aqueous media, have moderate toxic effects and provide mosquitocidal properties against the malarial vector *Anopheles stephensi*. The biosynthesized silver nanoparticles of this study are easy to prepare with high stability and provide pronounced toxic effects on target mosquito vectors. At the same time the nanoparticles used here enhance the feeding activities of mosquito fish in an environment that is nanoparticle contaminated to feed on target mosquito larvae. The present paper also revealed that physiological stress extended to changed enzymological activity levels of the mosquito fish *G. affinis* in a nanoparticle treated environment.
